# Impact of ergot alkaloid and steroidal implant on whole-body protein turnover and expression of mTOR pathway proteins in muscle of cattle

**DOI:** 10.3389/fvets.2023.1104361

**Published:** 2023-04-18

**Authors:** Taylor D. Ferguson, Caroline M. M. Loos, Eric S. Vanzant, Kristine L. Urschel, James L. Klotz, Kyle R. McLeod

**Affiliations:** ^1^Department of Animal and Food Sciences, University of Kentucky, Lexington, KY, United States; ^2^Forage Animal Production Research Unit, Agricultural Research Service, United States Department of Agriculture, Lexington, KY, United States

**Keywords:** bromocriptine, estradiol plus trenbolone acetate, mTOR, protein turnover, insulin, cattle

## Abstract

**Introduction:**

Holstein steers (*n* = 32) were used to determine if the ergot analog, bromocriptine decreases muscle protein synthesis through inhibitory action on the mTOR pathway *via* a direct effect on signal proteins, and if these negative effects can be alleviated with anabolic agents.

**Methods:**

Steers were treated with intramuscular administration of bromocriptine (vehicle or 0.1 mg/kg BW) and a subdermal commercial steroidal implant containing trenbolone acetate (TBA) and estradiol 17β (with or without), in a 2×2 factorial design. During the 35 day experiment, intake was restricted to 1.5 times maintenance energy requirement. On days 27 through 32, steers were moved to metabolism stalls for urine collection, and whole-body protein turnover was determined using a single pulse dose of [^15^N] glycine into the jugular vein on day 28. On day 35, skeletal muscle samples were collected before (basal state) and 60 min after (stimulated state) an i.v. glucose challenge (0.25 g glucose/kg). Blood samples were collected at regular intervals before and after glucose infusion for determination of circulating concentrations of glucose and insulin.

**Results:**

Bromocriptine reduced insulin and glucose clearance following the glucose challenge, indicating decreased insulin sensitivity and possible disruption of glucose uptake and metabolism in the skeletal muscle. Conversely, analysis of whole-body protein turnover demonstrated that bromocriptine does not appear to affect protein synthesis or urea excretion. Western immunoblot analysis of skeletal muscle showed that it did not affect abundance of S6K1 or 4E-BP1, so bromocriptine does not appear to inhibit activation of the mTOR pathway or protein synthesis. Estradiol/TBA implant decreased urea excretion and protein turnover but had no effect on protein synthesis, suggesting that steroidal implants promote protein accretion through unchanged rates of synthesis and decreased degradation, even in the presence of bromocriptine, resulting in improved daily gains. Implanted steers likely experienced increased IGF-1 signaling, but downstream activation of mTOR, S6K and 4E-BP1, and thus increased protein synthesis did not occur as expected.

**Conclusions:**

Overall, this data suggests that bromocriptine does not have a negative impact on muscle protein synthetic pathways independent of DMI.

## 1. Introduction

The mutualistic relationship between the grass *Festuca arundinacea* and the endophyte fungus *Epichloё coenophiala* creates an exemplary forage for livestock that has been planted on millions of acres throughout the United States. The endophyte is given nutrition, protection, and an avenue for reproduction, while the plant receives advantages in persistence, survivability, and protection against biotic attack (1). One of these endophytic protections is the production of ergot alkaloids in the tissues of tall fescue. These compounds have been determined to cause the variety of symptoms present in animals affected by fescue toxicosis. The most prominent symptoms seen in cattle consuming endophyte infected fescue are reduced feed intake, lower weight gains, respiratory distress, vasoconstriction, and disruption of normal hormone levels (2).

Ergot alkaloids contain an ergoline ring, similar to the neurotransmitters serotonin, dopamine, norepinephrine and epinephrine, allowing them to bind to biogenic amine receptors and elicit negative effects (3, 4). These biogenic amines regulate appetite, cardiovascular function, endocrine activity, gastrointestinal motility, muscle contraction, and thermoregulation in mammals (4, 5). The classic indication of fescue toxicosis is a significant reduction in circulating prolactin resulting from agonistic action of ergot alkaloids on D2 dopamine receptors (6). Prolactin is essential for regulating metabolism, the immune system, milk synthesis, milk secretion, and pancreatic development. Cattle suffering from fescue toxicosis have prolactin levels that are too low to trigger seasonal shedding. Low prolactin may also cause seasonal breeding livestock species to have reduced reproductive efficiency (3, 7).

In relation to nutrient metabolism, modified endocrine function and hormone secretion may also play a role in decreased livestock productivity. The synthetic ergot alkaloid bromocriptine is known to activate D2 receptors and block D1 receptors, as well as cause partial agonism of 5-HT2B serotonin receptors and inhibition of 5-HT2A receptors, resulting in reduced blood glucose and serum triglycerides, and decreased BW (8, 9). Bromocriptine also directly activates the α2 adrenergic receptors, inhibiting glucose-stimulated insulin secretion in pancreatic beta cells (8, 9). Intravenous administration of an ergotamine bolus to cows has also been shown to decrease plasma insulin concentrations and increase glucagon within 1 h of dosing (10). The exact mechanism of action of these ergot alkaloids on the pancreas is not clear.

One attempt to improve the performance of stocker cattle grazing endophyte infected tall fescue is the use of steroidal implants, which have been shown to increase ADG in animals eating infected pasture (11–14). Androgenic and estrogenic steroids have been used for decades to enhance feed efficiency, rate of gain, and muscle gain in feedlot cattle. Combined treatment of cattle with estradiol and trenbolone acetate results in an increased number of muscle satellite cells, increased expression of IGF-1 mRNA in muscle tissue, and increased levels of circulating IGF-1 (15). It is currently believed that local production of IGF-1 in skeletal muscle plays a role in supporting normal muscle growth and that it is at least partially responsible for the increased number of satellite cells, increased hypertrophy and increased muscle growth observed in anabolic steroid-treated animals (16). However, Davenport et al. (17) demonstrated that cattle grazing endophyte-infected pastures not only had lower rates of gain compared to controls, but also exhibited a slight decrease in IGF-1 responsiveness to estradiol-17β.

IGF-1 and insulin are also important stimulators of the mammalian target of rapamycin (mTOR) pathway, which in turn is responsible for protein synthesis and muscle gain in growing animals (18). Ergot alkaloids and structurally similar chemicals (indoles) have been shown to inhibit both the growth hormone axis when given by intraperitoneal injection to rats (19) and the mTOR pathway in cancer cells (20). This led to the hypothesis that ergot alkaloids negatively affect the mTOR pathway and protein synthesis. The objective of the current study was to determine if the ergot analog, bromocriptine decreases muscle protein synthesis though inhibitory action on the mTOR pathway *via* a direct effect on signal proteins, and if these negative effects can be alleviated by implantation with anabolic agents.

## 2. Materials and methods

All procedures used in this study were approved by the University of Kentucky Institutional Animal Care and Use Committee. Experiments were conducted at the University of Kentucky C. Oran Little Research Center in the Intensive Research Building in Versailles, Kentucky.

### 2.1. Animals and housing

Thirty-two Holstein steers (initial BW 332.6 ± 54.6 kg) were used in a 6 week experiment to determine the effects of the synthetic ergot alkaloid, bromocriptine, on protein turnover and skeletal muscle mTOR pathway activation with or without the presence of a steroidal implant. Steers were housed indoors at thermo-neutral conditions (22°C) in individual 3 m x 3 m pens with *ad libitum* access to water. A 14 h:10 h, light:dark cycle was established with lights turning on at 0600 h and off at 2000 h each day. Animals were fed a corn silage-based diet (Table 1) fed to provide 1.5 x net energy for maintenance daily. This feeding regimen was chosen to minimize potential variation in feed intake across treatments while ensuring sufficient energy and nutrient intake to support muscle accretion. Feed and water intake were recorded daily for each animal. Steers were weighed weekly and feeding amounts were adjusted accordingly. All animals were adapted to housing and diets for 5 days prior to the start of the experiment (day 1).

**Table 1:** Composition of experimental diet fed to steers.

Item	Percentage (DM basis)
*Ingredient*	
Corn silage	61.00
Cracked corn	24.00
Supplement^1^	10.00
DDGS	5.00
*Analyzed chemical components*	
DM, %	49.00
Crude Protein, %	13.00
Ca, %	0.55
P, %	0.48
*Calculated energy densities*	
NE_m_, Mcal/kg	0.81
NE_g_, Mcal/kg	0.52

1 Supplement composition: soybean meal, ground corn, limestone, trace mineral premix, fat, vitamin premix, and Rumensin 90. The trace mineral premix provided 92.9% salt, 68 ppm Co, 1838 ppm Cu, 120 ppm I, 9290 ppm Mn, 19 ppm Se, and 5,520 ppm Zn. The vitamin premix supplied 1820 IU/kg Vitamin A, 363 IU/kg Vitamin D, and 227 IU/kg Vitamin E.

### 2.2. Experimental design and procedures

In a 35 day randomized complete block design, steers were blocked by BW and randomly assigned within block to one of four treatments arranged in a 2 × 2 factorial structure. Treatment factors consisted of a synthetic ergot alkaloid (bromocriptine) or carrier; and steroidal implant or no implant so that the four treatments were: (1) a negative control, CON (*n* = 8) no implant and injection with carrier only; (2) BROMO (*n* = 8), no implant and injection with bromocriptine; (3) IMP (*n* = 8), implant and injection with carrier only; (4) B + I (*n* = 8), implant and injection with bromocriptine. On day 1 of the experiment, animals in the IMP and B + I group received a commercial steroidal implant containing 120 mg of trenbolone acetate (TBA) and 24 mg of estradiol 17β (Revalor-S, Merck Animal Health, Kenilworth, NJ) that was placed subcutaneously in the ear. Animals assigned to the BROMO and B + I group, received intramuscular bromocriptine mesylate (Cayman Chemical, Ann Arbor, MI) injections every 3 days (days 1, 4, 7, 10, 13, 16, 19, 22, 25, 28, 31, and 34) at a rate of 0.1 mg/kg BW (21, 22). This injection protocol was based on a previous study comparing the bromocriptine treatment response with the response observed in cows fed endophyte-infected fescue seed; where bromocriptine given at this dosage elicited the same gene expression response in 90% of identified differentially expressed genes in mammary tissue (*n* = 866) (23). Bromocriptine was reconstituted as working stock (14.02 mg/mL in 95% ethanol) and diluted with saline so no more than 40% ethanol was given in a single injection. Carrier injections consisted of 95% ethanol diluted with saline (40 and 60%, respectively). Injection dose was adjusted weekly based on BW.

Weekly blood samples were obtained prior to feeding from each steer *via* jugular venipuncture into 10 ml vacutainer tubes with EDTA (Becton, Dickinson and Company, Franklin Lakes, NJ) and processed immediately (2000 x g for 10 min at 4°C). Plasma was harvested and stored at −20°C until further analysis. On days 27–32 of the experiment, steers were brought into individual 1.25 m x 2 m metabolism stalls for quantitative collection of urine to measure whole-body protein dynamics using isotope infusion with [^15^N] glycine as described below. On day 35, blood and obliquus externus abdominis muscle samples were collected before and after an intravenous glycemic challenge as described below.

### 2.3. Isotope infusion for whole-body protein turnover

On d 27, steers were fitted with an intravenous jugular catheter (MILA International, Inc., Florence, KY LA1415, 14 ga, 15 cm) and placed into individual metabolism stalls for measurement of whole-body protein turnover (d 27–32). Total urine output was collected by continuous suction using a rubber funnel system attached to the ventral portion of the abdomen, allowing urine to flow into a plastic collection vessel. Urine acidity was reduced to pH < 3 by adding 1 l of a 23.5% solution of H_3_PO_4_ to the collection vessel after each sampling to prevent ammonia nitrogen (*N*) loss and microbial growth. In order to determine background enrichment of ^15^N in urinary urea, urine was collected for approximately 15 h prior to isotope infusion; a representative aliquot was taken for later analysis. On day 28, a single pulse dose of [^15^N] glycine (98 atom percent excess; Cambridge Isotope Laboratories, Inc., Andover, MA) was given intravenously at 0730 h. The [^15^N] glycine infusate was prepared by dissolving [^15^N] glycine (3.0 mg/kg bodyweight) (24) in physiological saline (50 mL) and sterilizing by Millipore filtration (0.45 μM; Millipore, Bedford, MA) (25). After isotope administration, urine was sampled every 12 h, during which urine weight was recorded and representative aliquots (2% by weight) were taken at 12, 24, 36, 48, 60, 72, 84, and 96 h after administration of the pulse dose. Urine samples were kept separate for each collection time and stored at 0° C until analysis. Steers were returned to their individual pens upon collection completion on day 32. Jugular vein catheters remained in place and regularly flushed with heparinized saline to maintain patency until the biopsy procedures.

### 2.4. Muscle biopsies

On day 35, muscle biopsies were performed before and after a glycemic challenge to evaluate activation of mTOR pathway components. Briefly, the surgical site was aseptically scrubbed and locally anesthetized by injecting bupivacaine (5.0 mg/mL; Covetrus, Dublin, OH) into the area using an “inverted L” block. Baseline blood (*t* = −15, 0 min) and muscle (*t* = 0 min) samples were collected before rapid intravenous infusion with 50% dextrose at a dose of 0.25 g glucose/kg BW. This glucose dose has previously shown to increase phosphorylation of mTOR pathways signaling proteins in dairy cows (26). Subsequent blood (*t* = 5, 10, 15, 20, 25, 30, 45, 60, 90, and 120 min) and muscle (*t* = 60 min) were collected following glucose bolus infusion. Muscle samples were obtained by making a vertical incision (approx. 3 cm long) through the skin of the *paralumbar fossa*, after which approximately 300 mg of muscle tissues was obtained from the *musculus obliquus externus abdominis* for each biopsy. The skin incision was surgically closed and monitored for the next few days. Animals received meloxicam (1 mg/kg BW; Covetrus, Dublin, OH) for 2 days and antibiotic ointment over skin incision sites.

All blood samples were collected into heparinized syringes and immediately placed on ice. Blood samples were then processed (2000 x g for 10 min at 4°C) to harvest plasma and stored at −20°C until further analysis. Muscle tissues were processed for isolation of intracellular proteins immediately after collection as previously described (27). In brief, fresh muscle tissue (100 mg) was minced and placed in cold buffer solution (20 mM hydroxyethyl piperazineethanesulfonic acid, 2 mM ethylene glycol tetraacetic acid, 50 mM NaF, 100 mM KCl, 0.2 mM ethylenediaminetetraacetic acid, and 50 mM β-glycerophosphate) that contained a protease/phosphatase inhibitor cocktail (1:100 ratio with buffer; Cell Signaling Technology, Danvers, MA). Tissue samples were homogenized over ice at 15 s burst using a handheld homogenizer and subsequently centrifuged (10,000 × g for 10 min). The supernatant was then removed and stored at −80°C until further analysis.

### 2.5. Sample analyses for isotope enrichment

To prepare samples for measuring ^15^N enrichment of urinary urea, urine samples were first analyzed for urea concentration using a Technicon AutoAnalyzer II (SEAL Analytical Inc., Mequon, Wisconsin) (28). This was used to determine the volume of urine needed for each sample to contain 100 μmoles of urea during ion exchange clean up. Polypropylene cation exchange columns (Bio-Rad, Richmond, CA), filled with AG 50 W-X8 ion exchange resin (100–200 mesh, H+ form, Bio-Rad, Richmond, CA) were charged with NaOH and HCl, then used to isolate urea from ammonia. Urine samples were diluted with ddH_2_O to reach the desired urea concentration, then transferred to the charged columns. The first 5 ml of filtrate was discarded, then 20 mL of ddH_2_O was added to the column, and the eluent was collected into a scintillation vial. Vials were dried in a circulating air oven at 55°C for 3 days, then 2 mL 0.1 M phosphate buffer was added to each vial to solubilize the sample. 100 μL of reconstituted sample was then transferred to an Erlenmeyer flask containing 3 mL of phosphate buffer. A 5 × 25 mm strip of folded Whatman 934-AH filter paper with 30 μL of 2.5 M KHSO_4_ was placed in a stopper cup and attached to each flask. Then 0.2 mL urease type III (100 unit/mL, Millipore Sigma, St. Louis, MO) was injected into the flasks in order to convert urea N to NH_3_. Flasks shook for 1 h at room temperature. Subsequently, 0.2 ml 3 N NaOH was injected into the flasks to volatilize NH_3_, and then shook again for 1 h at room temperature. After resting 24 h, stopper units were placed in a desiccator with an open container of concentrated H_2_SO_4_ and allowed to dry for 24 h. Then filter paper was enclosed in tin capsules and submitted for commercial analysis of ^15^N enrichment (UC Davis Stable Isotope Facility, Department of Plant Sciences, Davis, California) by automated mass spectrometry.

### 2.6. Calculation of protein turnover

The “[^15^N] glycine single dose urea end product method” was used to measure whole-body protein turnover (25, 29).

Total protein turnover was calculated as described previously (30) as:


Q=d/G

Where Q is protein turnover (g N/d), d is the rate of urea N excretion (g/d) in urine on day 28, 29, and 30, and G is the fractional recovery (%) in urinary urea of ^15^N from [^15^N] glycine. The ^15^N enrichment of each urine sample, following the pulse dose, was corrected by subtracting background ^15^N enrichment.

Whole-body protein synthesis was calculated as:


PS=Q−Nexcretion

Where PS is protein synthesis (g N/d), Q is protein turnover (g N/d), and *N* excretion is the amount of urea *N* excreted in urine.

### 2.7. Western immunoblot analysis

Muscle samples were analyzed by western immunoblotting to determine the relative abundance of total and phosphorylated proteins associated with the regulation of muscle protein synthesis (i.e., mTOR, p70 S6 Kinase (p70S6K), and eukaryotic translation initiation factor 4E-binding protein 1 (4E-BP1)) as previously described (27). Briefly, total protein content of sample homogenate was determined using a Bradford assay (Bradford Reagent; VWR International, Radnor, PA), after which samples were diluted to a final protein concentration of 4 μg/μL in Laemmli buffer (Bio-Rad Laboratories, Hercules, CA). Proteins were separated by sodium dodecyl sulfate (SDS)-PAGE by loading 40 μg of protein per sample, into 8% (mTOR/p70S6K) or 12% (4E-BP1) polyacrylamide gels. Electrophoresis was run at 100 V for 20 min, and then 180 V until the marker reached the bottom of the gel. Proteins were then transferred to a 0.45 μm polyvinylidene deflouride membrane for immunoblotting (Millipore Sigma, St. Louis, MO). Rabbit monoclonal antibodies were used against total (1:1000 diluted in a 5% (w/v) bovine serum albumin (BSA) tris-buffered saline with 0.1% Tween (TBST) solution, #2983, Cell Signaling Technology, Danvers, MA) and Ser2448 phosphorylated (1:1000 diluted in BSA/TBST solution, #5536, Cell Signaling Technology, Danvers, MA) mTOR, total (1:1000 diluted in 5% fat free milk/TBST solution, #2708, Cell Signaling Technology, Danvers, MA) and Thr389 phosphorylated (1:1000 diluted in 5% fat free milk/TBST solution, #9234, Cell Signaling Technology) S6K and total (1:1000 diluted in milk/TBST solution, #9644, Cell Signaling Technology, Danvers, MA) and Thr37/46 phosphorylated (1:1000 diluted in 5% fat free milk/TBST solution, #2855, Cell Signaling Technology, Danvers, MA) 4E-BP1. A chemiluminescent kit (GE Healthcare Bio-Sciences, Pittsburgh, PA) and digital imager (Azure biosystems, Dublin, CA) were used to visualize and capture protein images.

All antibodies were validated to determine specificity against mTOR, p70 S6K and 4E-BP1 in bovine muscle tissues by use of blocking peptides. In brief, the primary antibody was mixed with a blocking peptide for each respective antibody (Cell Signaling Technology, Danvers, MA) in an increasing ratio of 1:0, 1:0.05, 1:0.5, and 1:5 antibody to antigen ratio diluted in 5% BSA or milk. The solutions were then incubated for 1 h at room temperature to allow antibody–antigen interaction. Next, 4 membranes containing identical samples were incubated with one of the 4 solutions and incubated at 4°C overnight and imaged the following day. Primary antibody specificity for bovine mTOR, p70 S6K and 4E-BP1 was confirmed as the chemiluminescent signal disappeared when increasing amount of blocking peptide was added to the antibody mixture.

For each assay, all samples from one block were run at the same time in duplicate gels to eliminate inter-assay variation. Data is expressed as the average of two gels. Band densities were quantified with a commercial densitometry software (AzureSpot, Azure biosystems, Dublin, CA) and expressed as relative abundance of the proteins in arbitrary units. To control for intra-assay variation, each sample was normalized to a positive control (pooled bovine muscle homogenate samples), which was loaded onto each gel. Additionally, to correct for possible gel loading errors, all band densities were normalized to the total amount of protein in each respective lane. Differences in total and phosphorylated mTOR, S6K and 4EBP-a proteins, as well as the ratio of phosphorylated to total abundance of each respective protein as a measure of activation, will be discussed.

### 2.8. Plasma prolactin, glucose, and insulin analysis

Plasma prolactin concentrations were measured in duplicate as described previously (31) using reagents for bovine prolactin provided by the National Hormone and Peptide Program (Dr. A.F. Parlow, Harbor-UCLA Medical Center, Torrance, Ca). Intra-and inter-assay coefficients of variation were 5.8 and 8.3%, respectively.

Plasma glucose concentrations were determined using a biochemical analyzer (Konelab 20XTi, Thermo Electron Corp., Waltham, MA). Intra-and inter-assay coefficients of variation were 1.1 and 3.6%, respectively. Plasma insulin levels were assayed using a commercially available radioimmunoassay kit (Porcine insulin RIA, Millipore Sigma) which had previously been used for analysis of bovine insulin and validated in our lab. All samples for each assay were run in duplicate and within the same day. Insulin intra-assay coefficient of variation for low and high quality controls was 2.2 and 3.13%, and inter-assay coefficient of variation for low and high quality controls was 4.65 and 4.66%, respectively.

### 2.9. Statistical analyses

Data were analyzed using mixed or glimmix procedures of SAS (version 9.4; SAS Institute, Cary, NC). Bromocriptine, implant, and their interaction were considered fixed with block as a random factor in the model. When fixed effects were significant, least square means were compared using Fisher’s LSD. Area under the response curve (AUC) was determined for prolactin, insulin and glucose using GraphPad Prism (v. 5.01, San Diego, CA). Area under the curve, time to peak and peak plasma concentrations were analyzed for insulin, glucose, and prolactin. Plasma prolactin concentration was determined from weekly blood samples taken over 29 days, with data for each day analyzed as a randomized complete block, terms of the model included bromo, implant, and their interactions, in a 2×2 factorial structure. The response of protein expression to glucose infusion determined by western immunoblot was analyzed as the difference between baseline (pre-infusion) values and post-infusion (60 min) values of each dependent variable. Post-infusion values were also analyzed using each animal’s baseline values as covariates to determine if pre-infusion values affected post-infusion values and if this could be influenced by treatment. For covariate analysis, independent slope models were evaluated by testing baseline values (pre) and interactions between baseline values and treatment as fixed effects (pre, pre x bromo, pre x implant, and pre x bromo x implant) to determine which covariates were significant (*p* < 0.05). When interactions were not significant, interaction terms were dropped to evaluate common-slope covariate models. Nonsignificant covariates were dropped from the model. Graphs were created with GraphPad Prism (v. 5.01, San Diego, CA).

Model assumptions were assessed by evaluation of homogeneity of variances and normality of studentized residuals. For each data set, the homogeneous and heterogeneous models were compared for best fit using the BIC criterion. The model with the lowest BIC was preferred. Then, normality of the data was assessed by visual inspection of the distribution of studentized residuals, Q/Q plots, and a Shapiro–Wilk test (*p* < 0.05). Observations that disrupted normal distribution, with studentized residuals ≥3, were considered outliers and were removed from the datasets to obtain normal distribution. Four animal observations (two from CON, one from IMP, and one from B + I) were removed from the prolactin dataset based on distribution of residuals (all were over 3) and visual assessment of the data. One animal observation (BROMO) was removed from the weight gain dataset for d 8–15 based on a residual over 3 and weight gain that was unusually high from visual observation of the dataset. Seven animal observations (one from CON, two from BROMO, two from IMP, and two from B + I) were removed from the protein metabolism dataset based on urine collection errors and visual observation of the data. One more animal observation (B + I) was removed from the dataset based on residuals that were over 3 and irregularly high urea excretion and fractional recovery, which affected calculation of protein turnover and synthesis. Lastly, four animal observations were removed from the western immunoblot datasets based on distribution of residuals. The following observations, with residuals over 3, were removed: one from activation status, pre-infusion mTOR (CON), one from phosphorylated, post-infusion mTOR (IMP), one from activation status, pre-infusion S6K (IMP), and one from total, post-infusion S6K (BROMO).

## 3. Results

Throughout the 35-day experiment, DMI was restricted to 1.5 times maintenance energy requirement. Accordingly, DMI was the same for all treatments (*p* ≥ 0.22; Table 2). The average BW at enrollment was 333 ± 54.6 kg and did not differ between treatment groups (*p* ≥ 0.29). Implanted steers tended (*p* = 0.07) to have higher gains during the first week, but bromocriptine did not influence (*p* = 0.79) gain. Treatment differences were not significant until week two, during which weight gain was greater (*p* = 0.01) in implanted animals and less (*p* = 0.04) in bromocriptine treated animals. During the third week, implanted steers had higher (*p* = 0.02) gains, while those receiving bromocriptine showed no differences in gain (*p* = 0.12). Implants increased total gain and ADG (*p* < 0.0001) regardless of bromocriptine administration, and bromocriptine administration decreased gain (*p* = 0.01), independent of implant effects (interaction *p* ≥ 0.15).

**Table 2:** DM intake, initial BW, ADG, and prolactin area under the curve in steers treated with bromocriptine and estradiol/TBA implants.

	+ Bromo		− Bromo			*p*-values		
	+ Implant	− Implant	+ Implant	− Implant	SEM^1^	Bromo	Implant	Bromo^*^Implant
DM Intake, kg/day	6.18	6.14	6.24	6.20	0.08	0.22	0.44	0.99
Initial BW, kg	330.9	329.9	334.3	335.1	5.6	0.29	0.99	0.82
Weight change d1-8, kg	5.68	4.66	7.61	3.47	1.88	0.79	0.07	0.26
Weight change d8-15, kg	6.31	1.50	7.05	5.51	1.19	0.04	0.01	0.15
Weight change d15-22, kg	7.22	5.80	9.21	6.42	1.19	0.12	0.02	0.41
Total gain, kg	19.21	13.58	23.87	15.40	2.04	0.01	<0.0001	0.25
ADG, kg	0.92	0.65	1.14	0.73	0.10	0.01	<0.0001	0.25
Prolactin AUC	1853	1754	3013	3543	492	0.002	0.61	0.45

1 SEM, standard error of the mean. Data are presented as least squares means; +Bromo/+Implant (B + I) *n* = 8; +Bromo/-Implant (BROMO) *n* = 8, except for Weight change d8-15 value where *n* = 7; −Bromo/+Implant (IMP) *n* = 8; and −Bromo/−Implant (CON) *n* = 8.

### 3.1. Plasma prolactin

**Figure 1:**
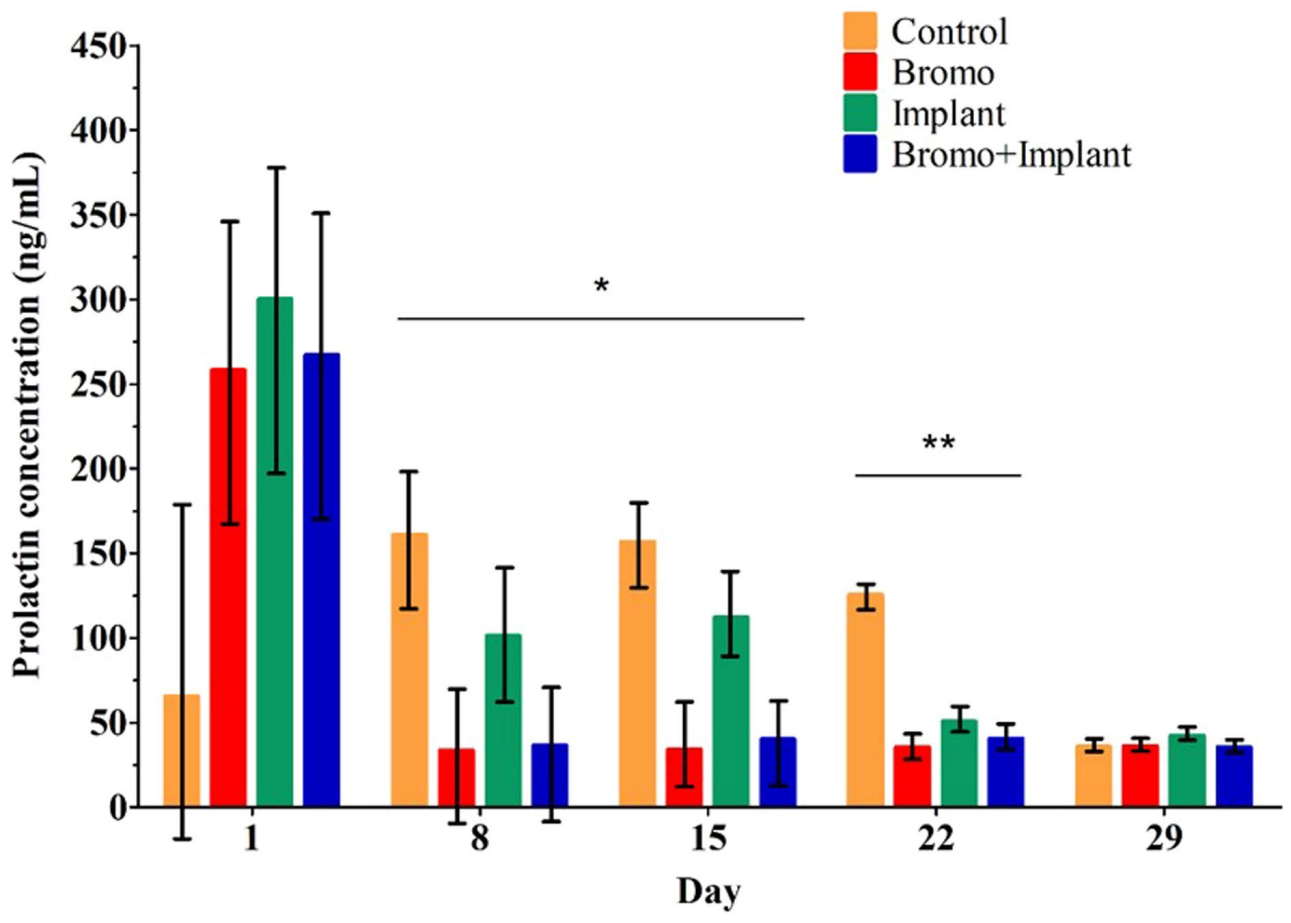
Mean plasma concentrations of prolactin (ng/mL). Values are means ± SE of plasma prolactin concentrations for steers treated with bromocriptine injections and estradiol/TBA implants from weekly samples over 29 days. Day 1 represents sample taken immediately before treatment administration. Days denoted with ^*^show bromocriptine treatment significantly decreased prolactin (Day 8, *p* = 0.04; Day 15, *p* = 0.001). Days denoted with ^**^show bromocriptine treatment significantly decreased prolactin (*p* = 0.002), implantation significantly decreased prolactin (*p* = 0.02), and a bromo x implant interaction (*p* = 0.01). Data for each day analyzed as a randomized complete block, terms of the model included bromo, implant, and their interactions, in a 2×2 factorial structure. Control *n* = 6: Bromo *n* = 8; Implant *n* = 7; and Bromo+Implant *n* = 7.

Weekly plasma prolactin concentrations are shown in Figure 1 and area under the curve measuring prolactin concentration over time are shown in Table 2. Before treatment began, there were no differences in circulating prolactin between groups (*p* ≥ 0.24) and there were no interactions. Steers that received bromocriptine injections experienced a sharp drop in prolactin after the first week of treatment (Day 8, *p* = 0.04) and maintained those depleted levels during week two (Day 15, *p* = 0.001) and three (Day 22, *p* = 0.002) (Figure 1). Consequently, prolactin AUC in steers treated with bromocriptine (1,803 ± 347) was significantly lower (*p* = 0.002) than the AUC of steers that were not treated with the alkaloid (3,278 ± 367). Prolactin concentrations were not different in steers treated with implant during the first 2 weeks of treatment (Day 8 & 15, *p* ≥ 0.45) and there were no interactions (Day 8 & 15, *p* ≥ 0.34). However, during the third week of treatment, implanted steers had significantly lower concentrations of prolactin (Day 22, *p* = 0.02) and there was a treatment interaction (Day 22, *p* = 0.01). During the fourth week of treatment circulating prolactin was not different between treatment groups (Day 29, *p* ≥ 0.53) and there was no treatment interaction (Day 29, *p* = 0.49). Despite this, implant treatment had no effect (*p* = 0.61) on prolactin AUC and there were no treatment interactions (*p* = 0.45) associated with prolactin AUC.

### 3.2. Protein turnover

Whole-body protein metabolism determined by [^15^N] glycine infusion and urine collection are shown in Table 3. Urinary excretion of urea (g/d) was lower (*p* = 0.006) in steers treated with estradiol/TBA implants. In contrast, bromocriptine had no effect (*p* = 0.13) on urea excretion and there was no treatment interaction (*p* = 0.87). The fractional recovery of labeled glycine from urine 48 h after infusion, was lower (*p* = 0.03) in implanted steers, but no differences (*p* = 0.30) were seen in bromocriptine steers. There was no treatment interaction (*p* = 0.87). Protein turnover tended to be lower in steers given steroidal implants (131.7 ± 7.8 g N/d) than in those that did not receive an implant (141.9 ± 7.5 g N/d) (*p* = 0.10), but there was no effect (*p* = 0.43) of bromocriptine on turnover. However, this was not true regarding protein synthesis, which was unaffected (*p* ≥ 0.47) by either implant or bromocriptine treatment. Treatment interactions were not present for protein turnover or protein synthesis (*p* ≥ 0.32).

**Table 3:** Protein metabolism in steers treated with bromocriptine and estradiol/TBA implants.

	+ Bromo		− Bromo			*p*-values		
	+ Implant	− Implant	+ Implant	− Implant	SEM^1^	Bromo	Implant	Bromo^*^Implant
Urea excretion (g/day)	38.88	50.60	44.28	57.27	4.97	0.13	0.006	0.87
^15^N Fractional Recovery	0.13	0.18	0.15	0.18	0.02	0.30	0.03	0.64
Protein Turnover (g N/day)	132.3	136.7	131.1	147.1	8.6	0.43	0.10	0.32
Protein Synthesis (g N/day)	112.4	113.0	109.4	118.6	7.5	0.85	0.47	0.52

1 SEM = standard error of the mean; Data are presented as least squares means; +Bromo/+Implant (B + I) *n* = 5; +Bromo/-Implant (BROMO) *n* = 6; −Bromo/+Implant (IMP) *n* = 6; and −Bromo/−Implant (CON) *n* = 7.

### 3.3. Plasma glucose

**Figure 2:**
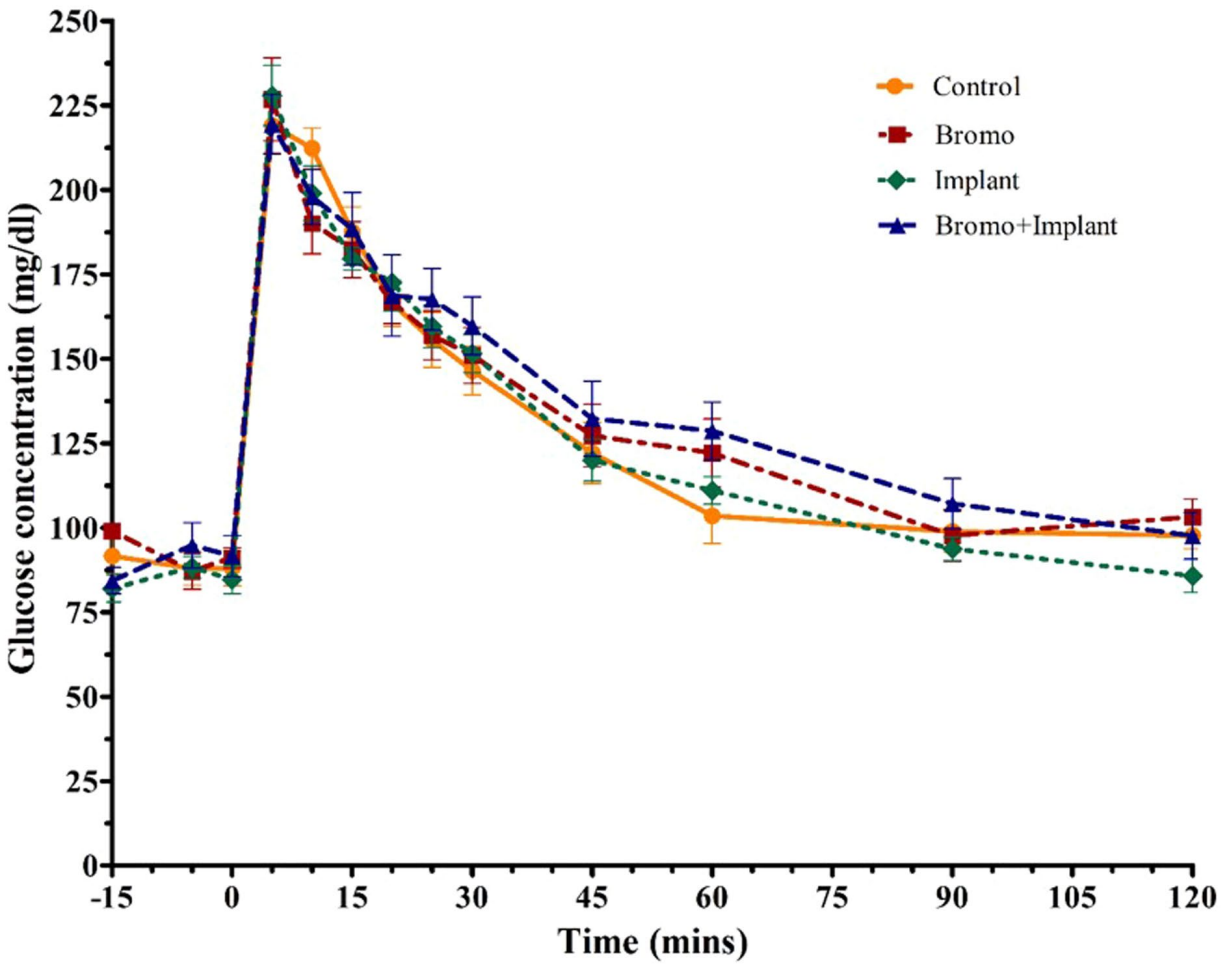
Mean plasma concentration of glucose (mg/dL). Values are means + SE of plasma glucose concentrations during intravenous glucose tolerance test (IVGTT) for steers treated with bromocriptine injections and estradiol/TBA implants performed on d 35. Used to determine area under the curve which tended to be higher with bromocriptine treatment (*p* = 0.09) and peak height. Time point 0 represents sample taken immediately before intravenous glucose bolus administration (0.25 g/kg BW). Area under the curve and peak height were analyzed as a randomized complete block, terms of the model included bromo, implant, and their interactions, in a 2×2 factorial structure. n = 8 for all treatments.

Plasma glucose concentrations in response to an intravenous glucose bolus are shown in Figure 2. The maximum concentration or peak height (Table 4) of blood glucose occurred at 5 min post infusion and was not different between treatment groups (*p* ≥ 0.70) and there was no treatment interaction (*p* = 0.32). There was a trend (*p* = 0.09) for AUC to be higher in steers treated with bromocriptine (17,155 ± 687 mg/dL) in comparison to steers not treated with bromocriptine (16,206 ± 687 mg/dl). Total AUC was not affected by steroidal implants (*p* = 0.73) and there was no treatment interaction (*p* = 0.45).

**Table 4:** Area under the curve and peak height of plasma glucose from steers treated with bromocriptine and estradiol/TBA implants before and following an IV glucose bolus.

	+ Bromo		− Bromo			*p*-values		
	+ Implant	− Implant	+ Implant	− Implant	SEM^1^	Bromo	Implant	Bromo^*^Implant
AUC	17450	16860	16095	16318	781	0.09	0.73	0.45
Peak Height (mg/dL)	219.5	231.0	228.2	223.0	8.8	0.97	0.70	0.32

1 SEM, standard error of the mean. Data are presented as least squares means; *n* = 8.

### 3.4. Plasma insulin

**Figure 3:**
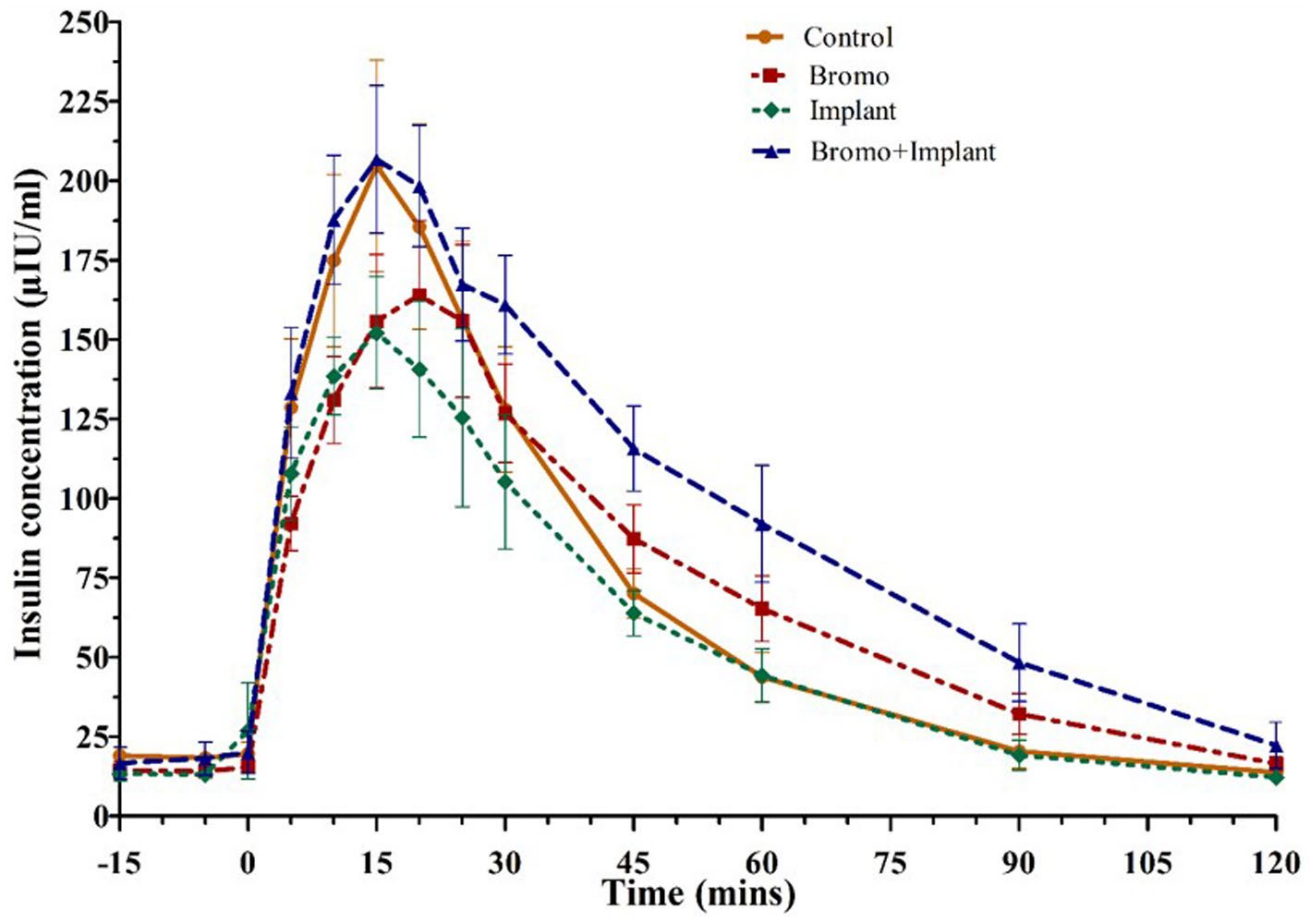
Mean plasma concentration of insulin (uIU/mL) Values are means ± SE of plasma insulin concentrations during intravenous glucose tolerance test (IVGTT) for steers treated with bromocriptine injections and estradiol implants performed on d 35. Used to determine area under the curve, peak height, and time to peak. Bromocriptine significantly increased AUC (*p* = 0.04) and there was a trend (*p* = 0.09) for it to increase time to peak. There was a significant bromo x implant interaction (*p* = 0.07) which increased AUC and peak height. Time point 0 represents sample taken immediately before intravenous glucose bolus administration (0.25 g/kg BW). Area under the curve, peak height, and time to peak were analyzed as a randomized complete block, terms of the model included bromo, implant, and their interactions, in a 2×2 factorial structure. *n* = 8 for all treatments.

Plasma insulin concentrations in response to an intravenous glucose bolus are shown in Figure 3. The time to peak insulin concentration (Table 5) was between 15-and 20-min post glucose infusion, and there was a trend (*p* = 0.09) for bromocriptine treated steers (18 ± 2 min) to take longer to reach peak insulin levels compared to steers that did not receive bromocriptine (15 ± 1 min). Time to peak was not affected by implant (*p* = 0.29) and there was no treatment interaction (*p* = 0.29). The maximum concentration (peak height) of insulin exhibited a tendency (*p* = 0.07) for a treatment interaction with no significant main effects (*p* ≥ 0.79). In the absence of bromocriptine, maximal concentration was lower for implanted steers, whereas in the presence of bromocriptine maximal concentration was higher for implanted steers. The insulin AUC of steers treated with bromocriptine (10,496 ± 818) was higher (*p* = 0.04) than that of untreated steers (8,052 ± 818). However, there was a tendency (*p* = 0.07) for a treatment interaction like that observed for peak height. In response to implant, insulin AUC was slightly decreased or unchanged in the absence of bromocriptine but was increased in the presence of bromocriptine.

**Table 5:** Area under the curve, peak height, and time to peak of plasma insulin from steers treated with bromocriptine and estradiol/TBA implants before and following an IV glucose bolus.

	+ Bromo		− Bromo		*p*-values		
	+ Implant	− Implant	+ Implant	− Implant	Bromo	Implant	Bromo^*^Implant
	Mean ± SE	Mean ± SE	Mean ± SE	Mean ± SE			
AUC	11,984 ± 1,136	9,005 ± 1,136	7,386 ± 1,136	8,718 ± 1,136	0.04	0.47	0.07
Peak Height (μIU/mL)	220.5 ± 26.2	167.8 ± 26.2	162.5 ± 26.2	211.3 ± 26.2	0.79	0.94	0.07
Time to Peak (mins)^1^	16.25 ± 2.89	20.00 ± 0.95	15.00 ± 1.61	15.00 ± 0.97	0.09	0.29	0.29

1 Data analyzed with heterogenous variance model.

Data are presented as least squares means ± standard error of the mean; *n* = 8.

### 3.5. mTOR Pathway

**Figure 4:**
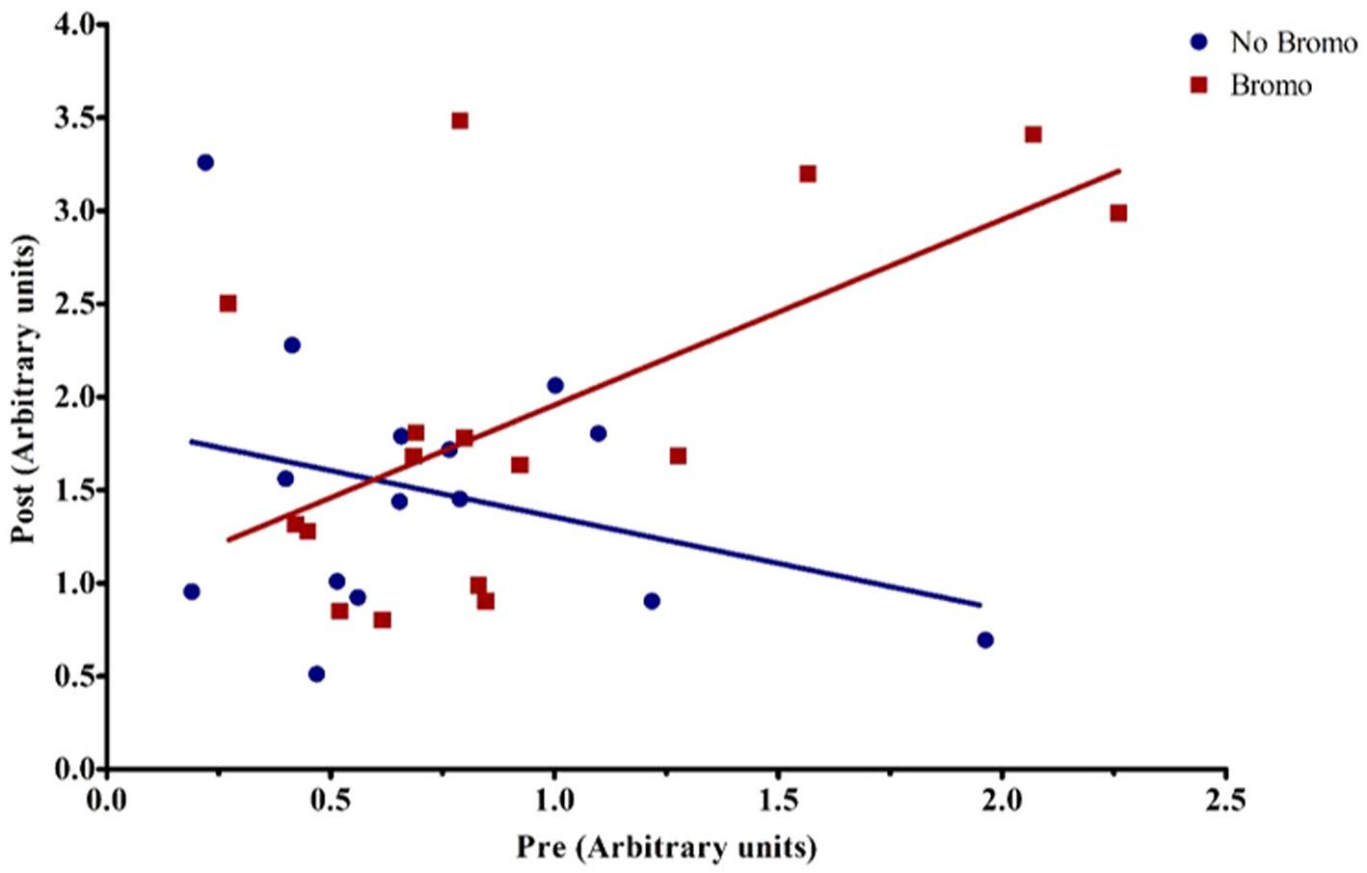
Representative graph of the interaction between pre-infusion levels of phosphorylated mTOR and bromocriptine treatment on post-infusion levels of phosphorylated mTOR. For covariate analysis, pre and its treatment interactions were first tested as fixed effects (pre, pre x bromo, pre x implant, and pre x bromo x implant) to determine which covariates were significant (*p* < 0.05). Insignificant covariates were dropped from the model. The pre x bromo interaction was significant (*p* = 0.0012), demonstrating that pre glucose infusion levels of phosphorylated mTOR affected the amount of phosphorylated mTOR found post infusion in animals treated with bromocriptine. There was a positive relationship between pre- and post- infusion abundance in bromocriptine treated steers (red fitted line). In the absence of bromocriptine (blue fitted line), there was no relationship between pre-and post-infusion phosphorylated protein abundance. Each point represents an individual steer.

Bromocriptine increased total abundance of mTOR protein (mTOR-T) in the basal state (*p* = 0.05; Table 6) and there was no effect of implant status (*p* = 0.55) or treatment interaction (*p* = 0.14). Neither basal levels of phosphorylated mTOR (mTOR-P) nor the activation status (phosphorylated mTOR protein:total mTOR protein) were affected (*p* ≥ 0.12) by implant, bromocriptine, or the interaction between these two (*p* ≥ 0.12). Basal abundance of mTOR-T affected (covariate: *p* = 0.01) post infusion values but not activation status (*p* = 0.96). Post- glucose infusion abundance of mTOR-T was not affected (*p* ≥ 0.20) by bromocriptine or implant treatment and there was no treatment interaction (*p* = 0.76). After glucose infusion, bromocriptine treatment increased abundance of mTOR -P (*p* = 0.04) with no effect on mTOR activation status (*p* = 0.38). The steroidal implant did not affect (*p* ≥ 0.14) mTOR-P abundance or activation status and there were no treatment interactions (*p* ≥ 0.42) for mTOR-P or activation status following glucose infusion. There was a covariate by bromocriptine effect on mTOR-P (*p* = 0.001). For steers receiving bromocriptine, there was a positive relationship between basal - and post- glucose infusion mTOR-P abundance. (Figure 4). However, in the absence of bromocriptine, there was no relationship between basal and post- infusion mTOR-P abundance.

**Table 6:** Relative abundance in arbitrary units of activated, phosphorylated, and total mTOR proteins in muscle of steers treated with bromocriptine and estradiol/TBA implants before (pre) and in response (post) to an IV glucose bolus.

		+ Bromo		− Bromo			*p*-values			Covariate *p*-values	
		+ Implant	− Implant	+ Implant	− Implant	SEM^1^	Bromo	Implant	Bromo^*^Implant	Pre	Pre^*^Bromo
Pre	Ratio	0.81	0.81	0.87	0.70	0.08	0.63	0.21	0.18	–	–
	Pho	0.81	1.07	0.84	0.64	0.18	0.19	0.83	0.12	–	–
	Total	0.97	1.23	0.90	0.79	0.13	0.05	0.55	0.14	–	−
Post	Ratio	1.35	1.28	1.25	1.27	0.10	0.38	0.69	0.53	0.98	0.96
	Pho	1.73	1.85	1.23	1.63	0.27	0.04	0.14	0.42	0.10	0.001
	Total	1.26	1.42	1.20	1.46	0.21	0.94	0.20	0.76	0.01	0.21

Ratio data represent the ratio of phosphorylated to total protein (i.e., activation status). Pho data represent the abundance of phosphorylated form of each protein. Total data represent the relative abundance of the total form of each protein.

1 SEM, standard error of the mean. Data are presented as least squares means; +Bromo/+Implant (B + I) *n* = 8; +Bromo/-Implant (BROMO) *n* = 8; −Bromo/+Implant (IMP) *n* = 8, except for Post-Pho values where n = 7; and −Bromo/−Implant (CON) *n* = 8, except for Pre-Ratio values where *n* = 7.

There was no effect (*p* ≥ 0.12; Table 7) of implant, bromocriptine, or their interaction on basal abundance of total (S6K1-T) and phosphorylated (S6K1-P) protein or activation status for S6K1. It was determined that basal abundance of S6K1-T affected (covariate: *p* < 0.0001) post infusion values but did not affect S6K1-P or activation status (*p* ≥ 0.17). Post glucose infusion abundance of S6K1-P and its activation status were unaffected (*p* ≥ 0.17) by any treatment. Post-infusion abundance of S6K1-T was unaffected (*p* = 0.40) by steroidal implant; however, abundance was lower (*p* = 0.05) for the bromocriptine treated steers compared with those not treated with bromocriptine. There were no (*p* ≥ 0.28) treatment interactions on SK61 post-infusion.

**Table 7:** Relative abundance in arbitrary units of activated, phosphorylated, and total S6K1 proteins in muscle of steers treated with bromocriptine and estradiol/TBA implants before (pre) and in response (post) to an IV glucose bolus.

		+ Bromo		− Bromo		*p*-values			Covariate *p*-values	
		+ Implant	− Implant	+ Implant	− Implant	Bromo	Implant	Bromo^*^Implant	Pre	Pre^*^Bromo
		Mean ± SE	Mean ± SE	Mean ± SE	Mean ± SE					
Pre	Ratio	0.08 ± 0.01	0.05 ± 0.01	0.07 ± 0.01	0.07 ± 0.01	0.82	0.24	0.12	–	–
	Pho^1^	0.10 ± 0.02	0.06 ± 0.01	0.10 ± 0.03	0.08 ± 0.02	0.63	0.12	0.63	–	–
	Total	1.32 ± 0.15	1.42 ± 0.15	1.34 ± 0.15	1.14 ± 0.15	0.20	0.60	0.15	–	–
Post	Ratio^1^	1.54 ± 0.55	0.63 ± 0.12	0.90 ± 0.36	0.75 ± 0.13	0.46	0.14	0.28	0.66	0.22
	Pho^1^	1.30 ± 0.54	0.53 ± 0.10	0.86 ± 0.37	0.67 ± 0.16	0.66	0.17	0.40	0.78	0.17
	Total	0.76 ± 0.06	0.76 ± 0.07	0.83 ± 0.06	0.92 ± 0.07	0.05	0.40	0.41	<0.0001	0.17

1 Data analyzed with heterogenous variance model.

Ratio data represent the ratio of phosphorylated to total protein (i.e., activation status). Pho data represent the abundance of phosphorylated form of each protein. Total data represent the relative abundance of the total form of each protein. Data are presented as least squares means ± standard error of the mean. +Bromo/+Implant (B + I) *n* = 8; +Bromo/-Implant (BROMO) *n* = 8, except for Post-Total values where *n* = 7; −Bromo/+Implant (IMP) *n* = 8, except for Pre-Ratio values where *n* = 7; and −Bromo/−Implant (CON) *n* = 8.

**Figure 5:**
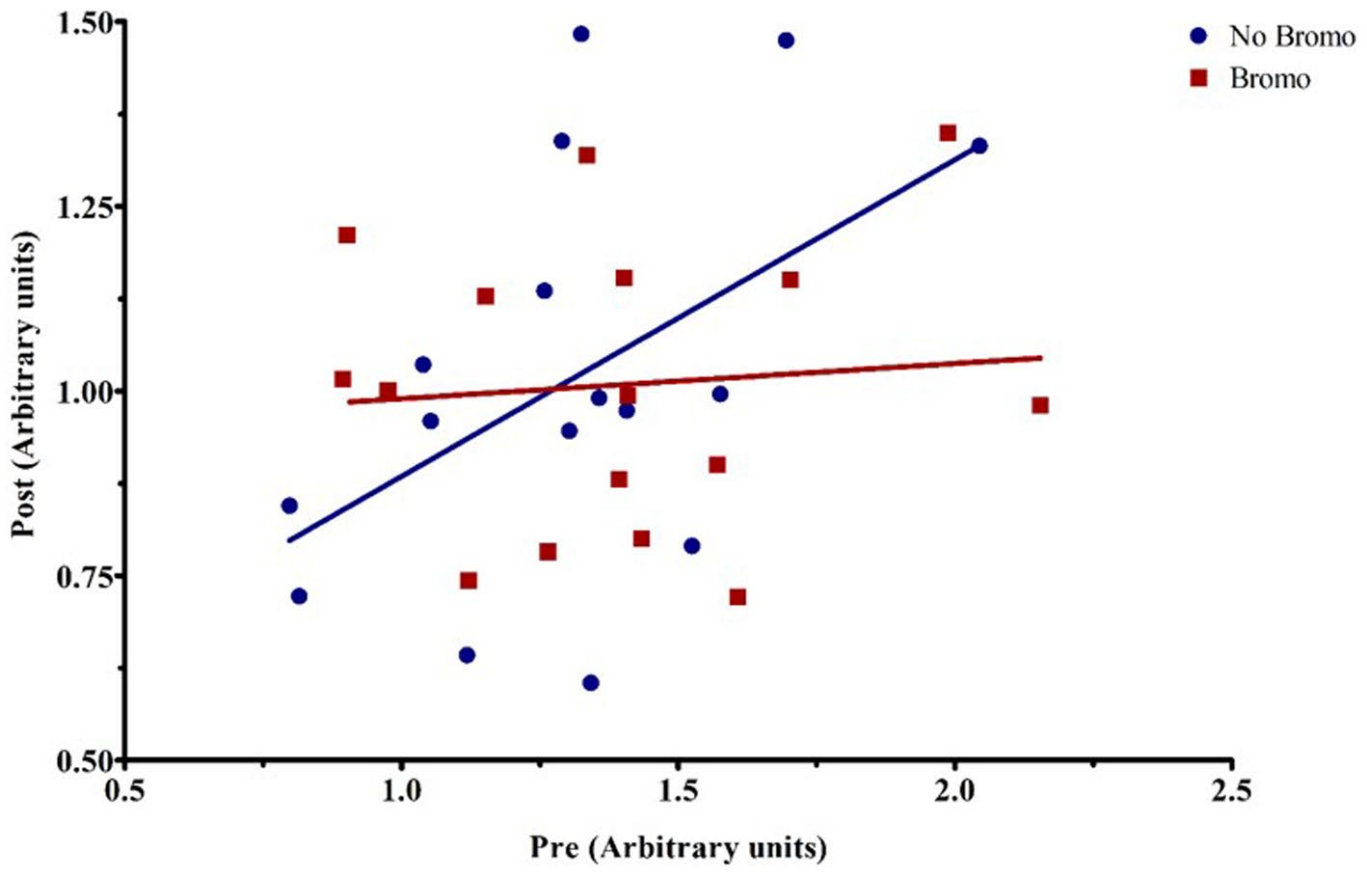
Representative graph of the interaction between pre-infusion levels of total 4E-BP1 and bromocriptine treatment on post-infusion levels of total 4E-BP1. For covariate analysis, pre and its treatment interactions were first tested as fixed effects (pre, pre x bromo, pre x implant, and pre x bromo x implant) to determine which covariates were significant (*p* < 0.05). Insignificant covariates were dropped from the model. The pre x bromo interaction was significant (*p* = 0.003), demonstrating that pre glucose infusion levels of total 4E-BP1 affected the amount of total 4E-BPI found post infusion in animals treated with bromocriptine. There was a positive relationship between pre-and post-infusion abundance in steers not treated with bromocriptine (blue fitted line), but in the presence of bromocriptine (red fitted line), there was no relationship between pre-and post-infusion total protein abundance. Each point represents an individual steer.

There was no effect (*p* ≥ 0.30; Table 8) of implant, bromocriptine, or their interaction on basal abundance of total (4E-BP1-T) and phosphorylated (4E-BP1-P) protein or activation status for 4E-BP1. However, there was a tendency for steroidal implant to increase the basal abundance of 4E-BP1-T in the absence, but not in the presence of bromocriptine treatment (bromocriptine-by-implant interaction, *p* = 0.06); there were no other treatment interactions for basal 4E-BP1 levels (*p* ≥ 0.15). Basal abundance of 4E-BP1-P affected (covariate: *p* ≤ 0.006) post infusion values but not activation status (*p* ≥ 0.3). Post-glucose infusion abundance of 4E-BP1-T was decreased by both bromocriptine (*p* = 0.005) and implant (*p* = 0.04). The lower total 4E-BP1 abundance for both implant and bromocriptine treatments, however, was largely explained by lower abundance for the implant treatment in the absence, but not in the presence of bromocriptine (bromocriptine-by-implant interaction, *p* = 0.10). Implant decreased abundance of post glucose infusion 4E-BP1-P (*p* = 0.02) and activation status of 4E-BP1 (*p* = 0.01), while bromocriptine had no effect on either (*p* ≥ 0.11). There were no treatment interactions (*p* ≥ 0.34) for post-infusion phosphorylated 4E-BP1 and activation status. There was a covariate by bromocriptine effect on 4E-BP1-T (*p* = 0.003). Without bromocriptine, there was positive relationship between basal and post glucose infusion values. With bromocriptine, basal values had little impact post glucose infusion 4E-BP1-T (Figure 5).

**Table 8:** Relative abundance in arbitrary units of activated, phosphorylated, and total 4E-BP1 proteins in muscle of steers treated with bromocriptine and estradiol/TBA implants before (pre) and in response (post) to an IV glucose bolus.

		+ Bromo		− Bromo			*p*-values			Covariate *p*-values	
		+ Implant	− Implant	+ Implant	− Implant	SEM^1^	Bromo	Implant	Bromo^*^Implant	Pre	Pre^*^Bromo
Pre	Ratio	0.71	0.58	0.58	0.62	0.08	0.42	0.38	0.15	–	–
	Pho	0.97	0.87	0.83	0.76	0.14	0.30	0.47	0.91	–	–
	Total	1.32	1.47	1.45	1.17	0.12	0.44	0.57	0.06	–	–
Post	Ratio	0.79	0.90	0.63	0.86	0.08	0.11	0.01	0.34	0.30	0.56
	Pho	0.74	0.90	0.66	0.90	0.10	0.60	0.02	0.59	0.006	0.80
	Total	0.99	1.01	0.96	1.15	0.09	0.005	0.04	0.10	<0.0001	0.003

Ratio data represent the ratio of phosphorylated to total protein (i.e., activation status). Pho data represent the abundance of phosphorylated form of each protein. Total data represent the relative abundance of the total form of each protein.

1 SEM, standard error of the mean. Data are presented as least squares means; *n* = 8.

## 4. Discussion

The purpose of this study was to use the ergot analog, bromocriptine as a model to determine if fescue-derived alkaloids affect whole-body protein metabolism and decrease muscle protein synthesis by directly inhibiting the mTOR pathway and whether an anabolic implant could mitigate this effect. A fescue toxicosis-like syndrome can be induced using bromocriptine injections and has previously been shown to decrease prolactin in steers and cows (21, 22). In one study, 90% of differentially expressed mammary genes (*n* = 866) responded similarly in bromocriptine-treated cows and those fed fescue-derived alkaloids (23) indicating that bromocriptine provides a satisfactory model for studying physiological changes in cattle affected by fescue derived alkaloids. Previous research has shown that anabolic steroids improve performance of cattle grazing endophyte infected fescue, though the exact underlying mechanism has not been fully elucidated (11–13). Steroidal implants are known to increase IGF-1 production in skeletal muscle, which is at least partially responsible for the increased muscle growth and weight gain observed in implanted animals (16) and could explain improved performance in cattle affected by ergot alkaloids. IGF-1 and insulin are important stimulators of the mTOR pathway, which regulates the initiation of protein synthesis and muscle gain in growing animals (18). Ergot alkaloids and structurally similar chemicals (indoles) have been shown to inhibit the growth hormone axis (19) and the mTOR pathway (20), in other species. This experiment is the first to demonstrate the effects of ergot alkaloids on the mTOR pathway in the skeletal muscle of cattle.

### 4.1. Plasma prolactin

In the current study, while prolactin appeared to decrease over time with all treatments (the exception being the increase from d1 to d8 for CON), prolactin in steers treated with bromocriptine decreased very sharply on d8 and stayed at nadir levels throughout the study — resulting in an overall decrease in area under the curve (AUC) for bromocriptine treated steers. Bromocriptine is a synthetic ergopeptine that activates dopamine receptors in the pituitary, and therefore imitates the actions of naturally occurring ergot alkaloids on prolactin secretion (32, 33). The bromocriptine effect was sufficient to be detected even against this background, indicating that a fescue toxicosis-like syndrome was induced. Despite this apparent decrease in the absence of bromocriptine, implant had no significant effect on area under the curve. The reason for this decrease is unclear and contrasts with previous reports where estradiol has stimulated prolactin release in male rats (34) and increased prolactin in the serum and milk of dairy cattle (35). Light is an important regulator of prolactin secretion, with shorter photoperiods decreasing prolactin (32). Before the experiment, animals were housed outside from April to July, with 12–14 h of light per day. During the experiment, the light:dark cycle was maintained at 14 h:10 h. Thus, light effects do not account for the aforementioned apparent decrease on circulating prolactin in the absence of bromocriptine.

### 4.2. DMI and weight gain

The diet was designed to meet the recommended nutrient intake of growing steers and intake was limited to 1.5x maintenance to eliminate confounding by potential treatment effects on DMI. This was important because ergot alkaloid consumption has been shown to decrease feed intake (21, 36). The agonistic action of alkaloids on serotonin receptors is thought to affect satiety and gastrointestinal motility (37, 38). Despite similar DMI, bromocriptine decreased ADG by approximately 16% over the 21-d period. This is consistent the with expected effects of fescue toxicosis (39) and previous reports which have shown that consumption of endophyte-infected fescue seed reduces VFA absorption (40) and the partial efficiency of ME use for retained energy in cattle (41). However, given the small difference in overall body weight gain (average of 3.24 kg) and the short duration of the monitoring period, it is unclear as to the impact of eating behavior, water consumption, or urine elimination may have had on our observations. Steroid implants are commonly used to enhance steer performance and have been shown to improve the gains of cattle grazing endophyte infected pastures (11–13), but this is one of the few instances where these effects have been studied with controlled DMI. Estradiol/TBA implant increased ADG both with (48% increase) and without (56% increase) bromocriptine, demonstrating that steroidal implants can improve weight gain, even when intake is controlled. The overall response to estradiol/TBA (52%) was greater than previously reported in studies with more observations and longer feeding periods in cattle fed at *ad libitum* 24–28% improvement; (42–44) or restricted intakes 14% improvement; (44, 45). Differences between the current study and those previously reported may reflect the short observation period and small number of animals used here.

### 4.3. Glucose homeostasis

Prior research has primarily focused on detrimental effects of ergot alkaloids on animal production and beneficial, growth promoting effects of hormonal implants, but little is known about the effects these have on glucose homeostasis in cattle. Bromocriptine has central and peripheral effects on the autonomic nervous system and the endocrine system in rats and humans (8). Its effects on pancreatic β-cells, and therefore glucose homeostasis, have made it an important treatment option for type 2 diabetes in humans, as it improves glucose tolerance and insulin sensitivity (8).

In the current study, an intravenous glucose tolerance test was used to activate the mTOR pathway and assess the first phase of insulin release. Interestingly, the insulin AUC was larger for bromocriptine treated animals and insulin concentrations took 30 min longer to return to baseline compared to untreated animals. Prolonged hyperinsulinemia could indicate there was a decrease in insulin sensitivity, which explains the tendency for glucose AUC to be higher in bromocriptine treated steers than in those not treated with bromocriptine. In lean mice, a single intraperitoneal injection of bromocriptine 1 h before an intraperitoneal glucose tolerance test has been shown to elevate blood glucose levels and reduced the rise in plasma insulin levels compared to mice that received a placebo, demonstrating acutely impaired glucose tolerance (46). Moreover, we have recently demonstrated that bromocriptine administration reduces insulin sensitivity in both healthy and insulin dysregulated horses (47). These results appear to contradict the improvements in glucose tolerance and insulin sensitivity seen in obese insulin resistant rodents and humans treated with bromocriptine (8). These conflicting results may be attributable to dose of bromocriptine, species differences, the use of healthy/lean animals instead of obese insulin-resistant animals or perhaps the timing of bromocriptine treatments.

Bromocriptine also tended to delay the rise in plasma insulin by 1.25 to 5.00 min in the absence of treatment effects on time to peak glucose concentrations; These results are in agreement with a previous study where ergot alkaloids decreased insulin and increased glucagon in heifers within 1 h of a single dose of ergotamine tartrate injection (10). The affinity of ergot alkaloids for the receptors of biogenic amines such as dopamine and norepinephrine in the pancreas may help explain the endocrine responses seen in bromocriptine treated animals. Dopamine type-2 receptors are expressed in the pancreatic β-cells of humans, rats, and mice, allowing dopamine to mediate inhibition of insulin secretion (48, 49). Dopamine decreases cell membrane depolarization and cytosolic Ca2+ entry, blunting insulin secretion in response to glucose stimulation (49). This could explain why D2-receptor agonists such as ergot alkaloids and bromocriptine have a similar effect on insulin secretion. Pancreatic β-cells also express α2-adrenergic receptors, which mediate the effects of norepinephrine released from the hypothalamus, postganglionic sympathetic nerve endings and the adrenal medulla (9, 50). Activation of these receptors by norepinephrine or α2-adrenoreceptor agonists such as bromocriptine leads to dysregulated glucose homeostasis and elevated blood glucose through potent inhibition of insulin secretion, increased glucagon secretion from the pancreas, and increased gluconeogenesis and glycogenesis in the liver (50).

Implanted steers tended to have increased peak insulin concentrations in the presence of bromocriptine but had lower concentrations in the absence of bromocriptine. This increase in maximal concentration, paired with an apparent decreased in insulin clearance, resulted in a tendency for B + I steers to have an insulin AUC that was higher than either treatment alone. Although we only measured insulin in the current study, it is well known that other hormones, including IGF-1 may be involved in regulating glucose homeostasis. Previous studies have shown steroid implants to increase IGF-1, which in turn could decrease insulin secretion (51–53). Elevated circulating IGF-1 binds to receptors on the surface of β-cells, activating a series of kinases that eventually activate the phosphodiesterase, PDE3B. This causes a reduction of cAMP levels in the β-cells and attenuation of insulin release from pancreatic islets (54). In humans, high levels of IGF-1 can directly stimulate glucose transport into muscle through either IGF-1 or insulin/IGF-1 hybrid receptors (55) which may explain why glucose clearance from the blood was not affected in implanted steers. There is limited research in cattle concerning the effects of the combination of ergot alkaloids on estradiol implants on circulating levels of IGF-1. Estradiol implants increased circulating IGF-1 in steers grazing pastures with low endophyte infection rates but not in steers grazing pasture with high endophyte infection rates (17). A single bolus injection of ergotamine tartrate in cattle has been shown to decrease plasma IGF-1 (56) and activation of D2 receptors by bromocriptine decreases growth hormone release in acromegalic patients, which inhibits GH-mediated antagonism of insulin (9). Paradoxically, ergotamine tartrate and ergonovine maleate have been shown to elevate plasma growth hormone concentrations in normal steers (57). Perhaps bromocriptine treatment removes the suppressive action of growth hormone and IGF-1 on insulin release, and therefore B + I animals show increased insulin secretion after the IVGTT. This does not account for all the effects on glucose homeostasis seen with combined bromocriptine and implant treatment. This result is likely due to a complex interplay of estradiol, growth hormone and IGF-1, in addition to bromocriptine and its activation of dopaminergic and adrenergic receptors in the pancreas. At this time, the exact mechanism of action for this novel response is unclear and requires further research to clarify what occurred in the current study.

### 4.4. Protein turnover

In the current study, a combined estradiol/TBA implant was used to maximize metabolic changes and growth response. The strong growth-promoting potency of TBA derives from both its anabolic activity as an androgen and anticatabolic activity as an anti-glucocorticoid. In cattle, estrogens are known to affect a variety of tissues to ultimately reduce nitrogen excretion, and improve mineral retention, protein anabolism and protein deposition in skeletal muscle (58). The exact mechanism of action involved for implant-stimulated muscle growth in beef cattle is still uncertain, but studies indicate their effects on muscle satellite cells may play an important role (16, 42, 59). It is currently believed that local production of IGF-1 in skeletal muscle plays a part in supporting normal muscle growth and an estradiol-induced increase in muscle IGF-1 mRNA expression contributes to the increased number of satellite cells, and increased rate and efficiency of muscle growth observed in anabolic steroid-treated animals (15, 16).

Steroidal implants appeared to increase N retention. Despite similar DMI among treatments in the current study, implants increased growth rate, and reduced urea excretion by 12 g/d, with no increase in protein synthesis rates and a trend for lower protein turnover rates, suggesting a decrease in protein degradation rate and an increase in *N* retention. This is in accordance with a previous study where predominant effects of implants (140 mg TBA/20 mg estradiol) in intake-restricted steers included decreased amino acid oxidation and increased nitrogen retention (60). Additionally, TBA has been shown to decrease protein synthesis and protein degradation in the muscle of female rats, but its effects on degradation seem more pronounced (61). Increased protein deposition through a decrease in protein degradation, as compared with an increase in protein synthesis, results in less energy expenditure for the animal.

DMI was restricted to eliminate confounding with intake effects, allowing observation of direct treatment effects on protein metabolism in tissues. Although total weight gain was decreased by bromocriptine in both the presence and absence of implantation, urea excretion was not affected by bromocriptine treatment. This is in accordance with previous studies (62, 63), although one study determined that N retention was lower in steers fed endophyte-infected hay with 13.8 g N/d vs. 22.4 g N/d in E-steers (63). It should be noted that DMI in those studies was not controlled to the extent of the current study, making comparisons difficult. Because fecal and urinary N were not affected by endophyte exposure in that report, reduced N retention was most likely a function of reduced intake (63). In the current study, bromocriptine did not affect whole-body protein synthesis or protein turnover. To the best of our knowledge, these aspects of protein metabolism in alkaloid-treated animals have not been documented in any species and suggest that the effects of ergot alkaloids do not stem from disruption of protein synthesis or degradation rates, *per se*.

It is unknown if ergot alkaloids affect production of IGF-1 in skeletal muscle or muscle growth induced by IGF-1, estradiol, and TBA in cattle. Considering bromocriptine did not have any effect on nitrogen excretion, protein turnover, and protein synthesis, the stunted weight gain in bromocriptine treated cattle may not be from an effect on protein accretion but rather glucose metabolism in skeletal muscle and protein degradation signals through the mTOR pathway.

### 4.5. mTOR signaling

The mTOR signaling pathway plays a central role in regulating cell growth and metabolism in eukaryotes through mediation by two multi-protein complexes, mTORC1 and mTORC2 (64). mTORC1 is an intermediary of nutrient and energy sensing and maintains the balance between anabolism and catabolism in response to environmental conditions. In the presence of pro-growth endocrine signals and sufficient energy, the mTORC1 pathway shifts toward increased anabolism and phosphorylates two substrates, S6K1 and 4E-BP1. Phosphorylation of S6K1 promotes protein synthesis and cell growth through multiple substrates, including translational regulator eIF4B, as well as eEF2K and ribosomal protein S6, both of which subsequently promote translation initiation and elongation (64–66). Multiple phosphorylations of 4E-BP1 by mTORC1 cause the protein to dissociate from eIF4E, allowing assembly of the eIF4F complex and mRNA translation to occur (18, 64). A mTORC1-dependent shift toward anabolism can only occur when the appropriate endocrine signals and sufficient energy are present. Both insulin and IGF-1 are important for activating signaling proteins, PI3K/Akt, upstream of mTOR, through the insulin receptor (IR) and IGF-1 receptors (IGF-IR). In turn, IGF-1/IGF-1R signaling to Akt/mTOR has been shown to be crucial in promoting muscle hypertrophy, with muscle growth resulting from increased protein synthesis or decreased degradation in myofibers (67).

As previously mentioned, bromocriptine decreased insulin and glucose clearance, which indicated decreased insulin sensitivity. This could directly impact insulin-mediated signaling and downstream activation of mTOR pathway proteins. However, phosphorylation of mTOR at Ser2448 after glucose stimulation was higher, suggesting potential positive influence of bromocriptine on mTOR activation. While this effect did not translate downstream, bromocriptine did not inhibit signaling protein phosphorylation. These data suggest that bromocriptine did not have an inhibitory effect on activation of the mTOR pathway and consequently did not inhibit protein synthesis.

It was expected that an anabolic, steroidal implant would increase mTOR pathway activation through an increase insulin and IGF-1 signaling. However, there was no increase in mTOR or S6K1 phosphorylation in the basal or stimulated state for implanted steers. In fact, activation status and phosphorylation of 4E-BP1 were lower in implanted steers, indicating interference with skeletal muscle protein translation initiation in the presence and absence of bromocriptine. While the current study did not show any significant decreases in protein synthesis in implanted steers, others have demonstrated a slight decline in protein synthesis with steroidal implants (60). These results partially contradict previous research in mice and rats showing that IGF-1 activation of mTOR stimulates protein synthesis and muscle hypertrophy through S6K1 and 4E-BP1 (67). Numerically, S6K1 showed the anticipated increases in activation status, but implants had the opposite effect on 4E-BP1. A possible explanation is the fact that mTOR exhibits differing kinase activity toward S6K1 and 4E-BP1 (68). Additionally, the mTOR protein has multiple residues that can undergo phosphorylation (68). Perhaps in the current study, phosphorylation occurred at sites other than Ser2448, which could explain disparities between activation of mTOR and phosphorylation of downstream proteins. Finally, alternate pathways, independent of mTOR could stimulate protein synthesis, such as inhibition of GSK3β by Akt (67). It has also been demonstrated that the stimulatory effect of IGF-1 on cell proliferation is mediated through the MEK/ERK pathway (69, 70) and inhibition of protein degradation in myotubes through the PI3K/Akt/FoxOsa pathway (71). Since protein abundance of upstream regulators of mTOR and these independent pathways were not evaluated in the current study, further investigation is required to get an accurate representation of the effects of estradiol/TBA implants on protein metabolism in cattle.

To our knowledge the effect of long-term administration of bromocriptine on the mTORC1, S6K, and 4E-BP1 has not been previously studied in cattle. The current study indicated that bromocriptine influenced potential relationships between pre-and post-glucose infusion levels of phosphorylated mTOR and total 4E-BP1. Bromocriptine treatment appeared to permit expression of a positive relationship between pre-and post-infusion abundance of phosphorylated mTOR. In other words, bromocriptine stimulation of post-infusion phosphorylated mTOR was only seen in cases with high pre-infusion concentrations of that phosphorylated protein. This suggests that bromocriptine may encourage activation of the mTOR protein at Ser2448. Conversely, bromocriptine appeared to nullify the positive relationship between pre-and post-infusion abundance of total 4E-BP1, suggesting that bromocriptine may have some effect on the expression of the 4E-BP1 protein. These findings demonstrate that long term administration of bromocriptine affected signal transduction between glucose, insulin, and the mTOR pathway. At this time, the exact mechanism of action for this novel response is unknown and requires further investigation of upstream signaling to mTOR and mTOR independent pathways to elucidate responses observed in the current study.

## 5. Conclusion

Bromocriptine injection induced a fescue toxicosis-like syndrome in steers, and treatment with an estradiol/TBA implant improved the growth of cattle, in the presence or absence of bromocriptine. Bromocriptine reduced insulin and glucose clearance after an IVGTT, indicating decreased insulin sensitivity and possible disruption of glucose uptake and metabolism in the skeletal muscle. This suggests that ergot alkaloids are detrimental to growing cattle in terms of overall glucose homeostasis and energy metabolism. However, there were no detrimental effects of bromocriptine on protein synthesis or indicators of N retention. Implantation improved apparent N retention, decreased protein turnover, and had no effect on protein synthesis, suggesting that steroidal implants promote protein accretion through unchanged rates of synthesis and decreased degradation, which may allow for more energy to be available for growth, even in the presence of bromocriptine. Although previous research suggested that combined estradiol/TBA treatment would increase IGF-1 signaling in implanted steers, activation of mTOR and stimulation of protein synthesis through downstream activation of S6K1 and 4E-BP1 was not detected, and results suggest steroidal interference occurred at 4E-BP1. These findings support the assertion that steroidal implants increase protein accretion through decreased protein turnover and degradation, even in the presence of bromocriptine. Bromocriptine did not affect S6K1 or 4E-BP1 phosphorylation, and therefore does not appear to inhibit activation of the mTOR pathway or protein synthesis, which agrees with the results of the protein turnover analysis. This suggests that, when feed intake is controlled, the decreased performance in cattle exposed to fescue-derived ergot alkaloids stems from issues with glucose homeostasis and skeletal muscle metabolism, disruption of upstream signals in the mTOR pathway, or disturbances in pathways independent of mTOR. These results also suggest that many of the differences in animal performance seen in cattle affected by fescue toxicosis may be attributed to differences in feed intake.

## Data availability statement

The raw data supporting the conclusions of this article will be made available by the authors, without undue reservation.

## Ethics statement

The animal study was reviewed and approved by University of Kentucky Institutional Animal Care and Use Committee.

## Author contributions

KM and TF conception and design of research. TF, CL, and KM performed experiment and lab analyses. TF, CL, EV, and KM analyzed data and interpreted results of experiments. TF prepared figures and drafted manuscript. TF, CL, EV, KU, JK, and KM edited and revised manuscript. TF, CL, EV, and KM approved final version of manuscript. All authors contributed to the article and approved the submitted version.

## Funding

This project was supported by USDA-Agricultural Research Service National Program 101, Food Animal Production. The information reported in this paper is part of a project of the Kentucky Agricultural Experiment Station and is published with the approval of the Director. Mention of trade name, proprietary product, or specified equipment does not constitute a guarantee or warranty by the University of Kentucky and does not imply approval to the exclusion of other products that may be available.

## Conflict of interest

The authors declare that the research was conducted in the absence of any commercial or financial relationships that could be construed as a potential conflict of interest.

## Publisher’s note

All claims expressed in this article are solely those of the authors and do not necessarily represent those of their affiliated organizations, or those of the publisher, the editors and the reviewers. Any product that may be evaluated in this article, or claim that may be made by its manufacturer, is not guaranteed or endorsed by the publisher.
